# Type 1 diabetes diagnosed during pregnancy—an unusual but important challenge: a case series and review of literature

**DOI:** 10.3389/fmed.2025.1656833

**Published:** 2025-09-12

**Authors:** Amelia Caretto, Erika Pedone, Andrea Laurenzi, Filippo Bolamperti, Caterina Cellai, Federica Pasi, Serena Girardelli, Maria Teresa Castiglioni, Nicoletta Dozio, Marina Scavini

**Affiliations:** ^1^Diabetes Research Institute, IRCCS San Raffaele Scientific Institute, Milan, Italy; ^2^Vita-Salute San Raffaele University, Milan, Italy; ^3^Department of Obstetrics and Gynaecology, IRCCS San Raffaele Scientific Institute, Milan, Italy; ^4^Azienda Socio-Sanitaria Territoriale (ASST) della Brianza, Vimercate, Italy

**Keywords:** type 1 diabetes, pregnancy, hyperglycemia in pregnancy, diabetes autoantibodies, diabetes onset, autoimmune diabetes in adults, latent autoimmune diabetes in adults (LADA)

## Abstract

Hyperglycemia diagnosed during pregnancy is most commonly due to gestational diabetes mellitus (GDM), while a minority of cases are attributable to previously unrecognized type 2 diabetes mellitus (T2DM) or, more rarely, type 1 diabetes mellitus (T1DM). Although T1DM has traditionally been associated with onset during childhood or adolescence, recent evidence shows that nearly 40% of new T1DM cases occur after the age of 30 years, coinciding with the age at which many women in Europe begin pregnancy. This highlights the importance of considering T1DM in the differential diagnosis of hyperglycemia during pregnancy, given the relevant implications for the management and outcomes of these pregnancies. Diabetes-related autoantibodies have been detected in pregnancies complicated by GDM, with prevalence varying widely (from less than 1% to up to 18%), depending on population risk, assay, and antibody type. The most commonly detected autoantibodies in GDM are islet cell antibodies (ICA) and anti-glutamic acid decarboxylase antibodies (GADA), while the presence of multiple autoantibodies is much rarer. Women with hyperglycemia and diabetes-related autoimmunity display a peculiar clinical profile: they usually have a normal pre-pregnancy BMI, low insulin resistance, and require insulin therapy more often than antibody-negative patients with hyperglycemia. They are typically younger and are likely to have a family history of diabetes or other autoimmune diseases. Importantly, the presence, and especially the number, of positive autoantibodies is associated with an increased risk of progression to T1DM after pregnancy. Therefore, identifying autoimmune markers of beta-cell damage in pregnant women with hyperglycemia is critical for prognosis, tailored management, and appropriate follow-up.

## 1 Introduction

Hyperglycemia diagnosed in pregnancy is mainly due to gestational diabetes mellitus (GDM), which complicates 7 to 12% of pregnancies in Europe (1). In a relatively small proportion of cases, hyperglycemia is due to previously undiagnosed type 2 diabetes mellitus (T2DM) that manifests for the first time during pregnancy, while in very rare cases, it is due to type 1 diabetes mellitus (T1DM). Diagnosis of these rare cases can be challenging but is very important because of the implications for patient care during pregnancy and thereafter.

Although T1DM has long been considered a disease with onset in infancy and adolescence, recent use of genetic scores has shown that almost 40% of the incident cases of T1DM occur in individuals over the age of 30 years (2). The third and fourth decades of life are when close to half of all pregnancies occur in the European Union (EU). In fact, because of the progressive increase in the age at the delivery of the first child over time, according to Eurostat in 2019, in the EU, the mean age at first childbirth was 29.4 years, ranging from 26.3 years in Bulgaria to 31.3 years in Italy. Therefore, although rare, cases of hyperglycemia first diagnosed during pregnancy may be due to T1DM, reflecting either the clinical onset of the disease or the worsening of pre-clinical T1DM.

Determining which type of diabetes is behind any instance of hyperglycemia diagnosed during pregnancy is important for many diverse reasons. The management of blood glucose in a pregnant woman with type 1 diabetes is challenging, and the option of a glucose sensor or an insulin pump, both technologies recommended by current guidelines (3), may not be available for patients with either gestational or type 2 diabetes. Pregnant women with type 1 diabetes (as well as type 2 diabetes) should be referred to centers with specialized expertise in antenatal care and delivery for patients with pregestational diabetes because of the significantly increased risk of unfavorable maternal and fetal outcomes in these pregnancies.

We here report a series of women diagnosed with T1DM during pregnancy who were cared for at the Diabetes and Pregnancy Clinic of our institution over the last 10 years. The cases were heterogeneous in terms of gestational weeks (GW) at diagnosis, mode of diagnosis, and glucose tolerance after delivery. However, all of them during pregnancy were prescribed intensive insulin therapy using a basal/bolus regimen and a sensor for continuous glucose monitoring when available. Pregnancy outcomes will also be included.

## 2 Case 1

A 31-year-old white woman at 26 + 2 gestational weeks (GW) of her second pregnancy was referred to our Diabetes and Pregnancy Clinic for significant hyperglycemia (patient characteristics are shown in Table 1). Her family history was positive for type 2 diabetes (T2DM)—maternal grandfather and paternal grandmother, both treated with oral agents—and autoimmune diseases, including her mother’s hypothyroidism due to chronic autoimmune thyroiditis, her brother’s documented celiac disease, and her 5-year-old daughter’s recent diagnosis of juvenile idiopathic arthritis. The patient had vitiligo on her neck and left shoulder. At 9 GW, she was diagnosed with hypothyroidism due to chronic autoimmune thyroiditis and treated with levothyroxine at doses of 25 mcg and 50 mcg on alternate days.

**Table 1:** Characteristics of the patients.

	Patient n.1	Patient n.2	Patient n.3	Patient n.4
Previous history of GDM	Yes	No	No	No
IFG before 16 GW	No	Yes (FPG 5.72 mmol/l and 6.78 mmol/l)	Yes (FPG 6.16 mmol/l)	No
Familial history of Diabetes	Yes	Yes	Yes	Yes
Onset of T1DM	24 GW	16 GW	Immediately after miscarriage	24 GW
Age at onset	31	32	38	31
75 g OGTT at 16–18 GW Time 0′ Time 60′ Time 120′	Yes 4.7 mmol/l (84 mg/dL) 8.9 mmol/l (160 mg/dL) 6.1 mmol/l (109 mg/dL)	Yes 7.9 mmoL/L (143 mg/dL) - -	No	No
75 g OGTT at 24–28 GW Time 0′ Time 60′ Time 120′	Yes 7.6 mmol/l (137 mg/dL) 14.4 mmol/l (260 mg/dL) 14.4 mmol/l (259 mg/dL)	No	No	Yes 5.28 mmol/l (95 mg/dL) 7.56 mmol/l (136 mg/dL) 6.72 mmol/l (121 mg/dL)
HbA1c at the time of diagnosis	44 mmol/mol	42 mmol/mol	35 mmoL/L	34 mmol/mol
HbA1c at the time of delivery	32 mmol/mol	37 mmol/mol	N/a	N/a
C-peptide at the time of diagnosis	0.62 nmol/l	0.25 nmol/l	0.51 nmol/l	N/a
ß-cells autoimmunity	GADAb+ IA-2Ab+ ZnT8Ab+ AINS+ ICA+	GADAb+ IA-2Ab+ ZnT8Ab+ AINS− ICA+	GADAb+ IA-2Ab− ZnT8Ab− AINS− ICA−	GADAb+ IA-2Ab− ZnT8Ab− AINS− ICA−
Pre-gestation BMI	18.9 kg/m^2^	20.2 kg/m^2^	20.1 kg/m^2^	N/a
Weight gain at the time of diagnosis	+10 kg	+4 kg	N/a	N/a
Weight gain at the end of gestation	+12.8 kg	+18 kg	N/a	N/a
Daily insulin starting dosage	0.37 UI/kg	0.27 UI/kg	0.1 UI/kg	-
Daily insulin dosage at the time of delivery	0.43 UI/kg	0.49 UI/kg	N/a	-
GW at the time of delivery	39th	38th	Miscarriage	38th
Complications at the time of delivery	No	Yes	Miscarriage	No
Newborn body weight	3,670 g	3,350 g	N/a	3,670 g
Insulin treatment discontinuation after delivery	Yes	No	Yes	-
Time from delivery to insulin dependence	36 months	0 months	>36 months	5 years

GW, gestational week; GDM, gestational diabetes; T1DM, type 1 diabetes mellitus; IFG, impaired fasting glucose; FBG, fasting blood glucose; HbA1c, glycosylated hemoglobin; GADAb, anti-GAD antibodies; IA-2Ab, anti-insulinoma antigen 2 antibodies; ZnT8Ab, anti-zinc transporter 8 antibodies; AINS, anti-insulin antibodies; ICA, islet-cells antibodies; N/a, not available.

During her first pregnancy, the patient was diagnosed with gestational diabetes (GDM) according to the results of a 75-gram oral glucose tolerance test (OGTT), although no treatment was recommended. The detection of macrosomia by ultrasound prompted induction of labor at 38 GW, resulting in the delivery of a baby girl of 3,860 grams, who spent a few days in the neonatal intensive care unit because of jaundice.

**Figure 1:**
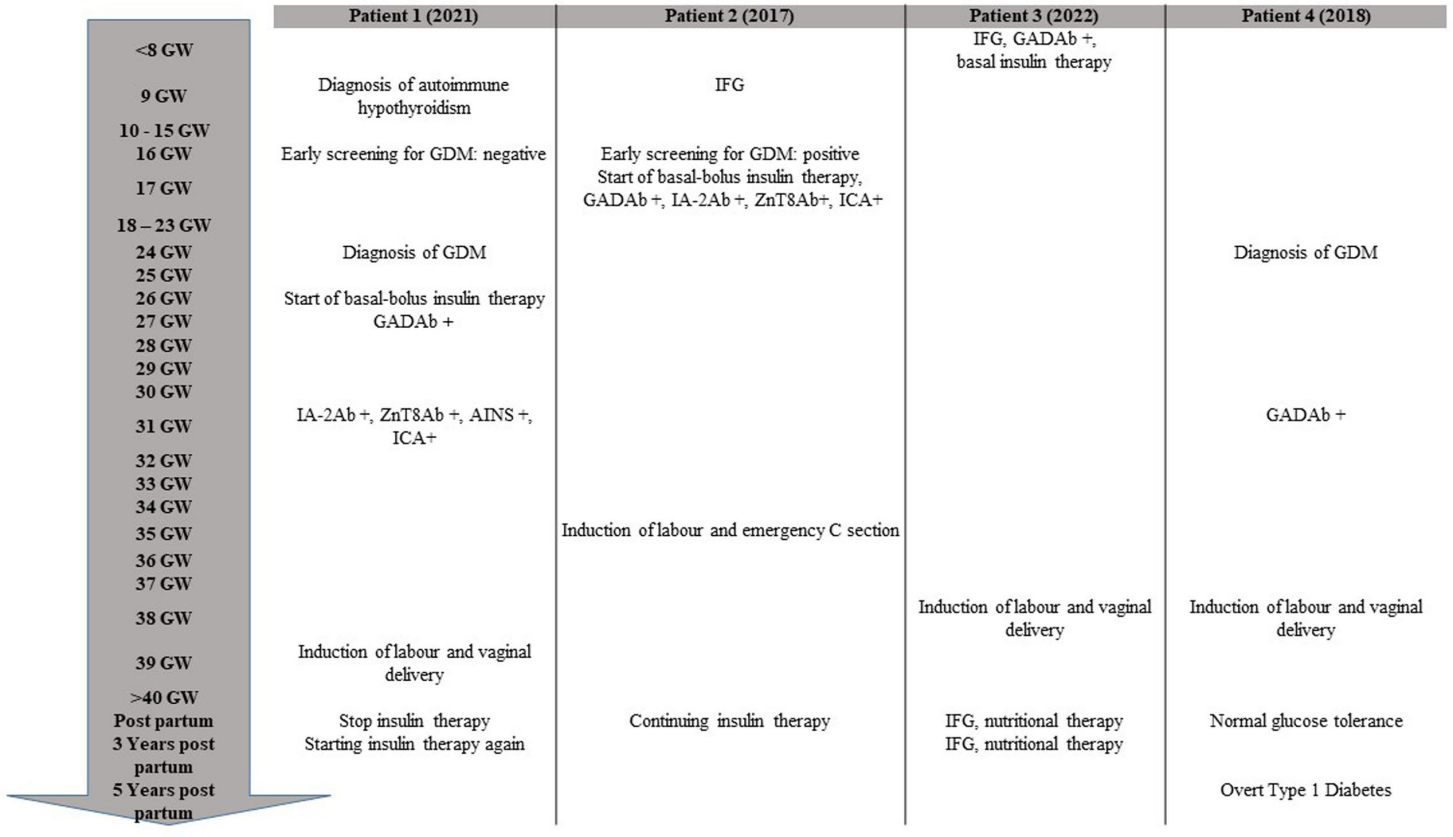
Timeline of chronological progression of disease during and after pregnancy. GW, gestational week; GDM, gestational diabetes; IFG, impaired fasting glucose.

During her second pregnancy, as recommended by the Italian guidelines (4) for the screening of GDM, the patient underwent a 75-gram OGTT at 16 GW, which was normal according to the IADPSG criteria (time 0’ BG 4.7 mmoL/L, time 60’ BG 8.9 mmoL/L, and time 120’ BG 6.1 mmoL/L) (timeline shown in Figure 1). A second 75-gram OGTT was then performed at 24 GW, documenting significant hyperglycemia (time 0’ BG 7.6 mmoL/L, time 60’ BG 14.4 mmoL/L, and time 120’ BG 14.4 mmoL/L) and prompting referral to our Diabetes and Pregnancy Clinic. Glycated hemoglobin was 44 mmol/mol (n.v. up to 42 mmol/mol). Her pre-pregnancy body weight was in the lower normal range (BMI 18.7 kg/m^2^), with a weight gain of 10 kg by 26 + 2 GW.

We provided the patient with education on diabetes management, including insulin injections and the use of a glucose sensor for continuous glucose monitoring. Unfortunately, due to supply issues from the local health care agency (Azienda Territoriale Sanitaria), she was not able to use it continuously. We started her on rapid-acting insulin prior to meals and snacks and long-acting insulin at bedtime, with an initial dose of 0.4 UI/kg per day, resulting in rapid normalization of blood glucose levels. Since the patient had a pre-gestational normal-low BMI (18.9 kg/m^2^) and markedly higher blood glucose values than expected in a case of GDM, we suspected the onset of T1DM. Beta-cell-specific autoantibodies were indeed present (anti-glutamic acid decarboxylase antibodies [GADA] > 250 U/mL, insulinoma antigen 2 [IA-2] 141 U/mL, zinc transporter 8 [ZnT8] > 500 U/mL, and islet cell autoantibodies [ICA] positive), and the fasting C-peptide level was low (0.62 nmoL/L).

Insulin dosage progressively increased to 0.43 UI/kg per day as the pregnancy advanced, maintaining optimal glucose control throughout the pregnancy and adequate weight gain (12.5 kg at term). The requirement for rapid-acting insulin was twice that of basal insulin, reflecting an insulin secretion deficiency. The patient gave birth at 39 GW to a healthy female baby who had no neonatal hypoglycemia (weight at birth 3,670 g, 81st centile of growth according to the INeS Chart, Apgar index 10 at 5′, 10 at 10′).

In the days following delivery, insulin treatment was gradually decreased and eventually stopped because of the detection of sustained near-hypoglycemic glucose levels. The patient maintained optimal glucose control without insulin treatment for nearly 36 months following delivery, after which blood glucose levels started to rise, necessitating the restart of insulin treatment. Fasting C-peptide had by then decreased to 0.39 nmoL/L, and HbA1c was 49 mmol/mol.

## 3 Case 2

A 32-year-old white woman was referred to our clinic at 9 GW of her first pregnancy due to evidence of impaired fasting glucose (first blood glucose report 5.72 mmoL/L, confirmed FBG 6.78 mmoL/L; patients’ characteristics are shown in Table 1).

A positive family history of diabetes and autoimmune diseases was reported. Her brother had a diagnosis of T1DM, and her maternal aunt was diagnosed with type 2 diabetes and chronic autoimmune thyroiditis. No relevant disease was reported in her medical history.

As recommended, the patient underwent a 75-gram OGTT at 16 GW, but the test was inappropriately halted when fasting blood glucose measured 6.77 mmoL/L (timeline shown in Figure 1). Due to pre-gestational normal BMI (20.2 kg/m2), limited weight gain at 16 GW (4.5 kg), and her family history of T1DM, we recommended testing for beta-cell-specific autoantibodies, which returned positive (GADA > 250 U/mL, ZnT8 Ab 324 U/mL, ICA positive). Fasting C-peptide levels were indeed very low (0.25 nmoL/L).

Promptly, after education on diabetes self-management, the patient was started on rapid-acting insulin analogs before meals at a dose of 0.27 UI/kg per day, with the addition of an intermediate-long-acting analog at approximately 60% of the rapid-acting insulin dose. A continuous glucose monitoring device was offered to the patient, but she preferred self-monitoring of blood glucose (SMBG). Glycated hemoglobin was 42 mmol/mol. Glycemic control was challenging due to high glucose variability and frequent hypoglycemic episodes, especially upon awakening, after meals, and following physical exercise. Insulin dosage progressively increased to 0.49 UI/kg per day, and HbA1c values reached 37 mmol/mol at term. Her body weight had increased by 18 kg at term.

Since the amniotic fluid index was reported in the lower-normal range (AFI 10 cm) during the ultrasound scan at 35 GW, the patient was closely monitored until 38 GW when labor was induced. Due to a non-reassuring fetal heart rate and a high fetal distress suspicion during induction, an emergency cesarean section was performed. The newborn was a healthy female baby, without jaundice, hypoglycemia, or other clinical complications during the neonatal period (weight at birth 3,350 g, 81st centile of growth according to INeS Chart, Apgar index 8 at 5’, 10 at 10’).

In contrast to case 1, the patient could not discontinue insulin treatment after delivery. During the years following delivery, she experienced periods of high glucose variability with poor glycemic control, but at her most recent visit, 8 years after her T1DM diagnosis, her glucose control was good (HbA1c 51 mmol/mol). Her BMI had increased to 25.7 kg/m^2^, and her total daily insulin dose was 0.3 UI/kg.

## 4 Case 3

A 38-year-old white woman was referred to our clinic because of miscarriage during her first pregnancy at 6 + 2 GW and concurrent evidence of impaired fasting glucose (fasting blood glucose 6.16 mmoL/L, see Table 1 for patient characteristics). Her family history revealed no susceptibility to autoimmune diseases, but her paternal uncle had T2DM. No relevant diseases were reported in her medical history. Glycated hemoglobin was 35 mmol/mol. Beta-cell-specific autoimmunity and thyroid function were determined since impaired fasting glucose is usually uncommon within the first 15 weeks of gestation in patients with a BMI in the lower-normal range (20.1 kg/m2). Moreover, the patient was seeking a new pregnancy. GADA were positive at a mid-low titer (11 U/mL) (5), and concomitant subclinical autoimmune hypothyroidism was diagnosed (TSH 4.08 mU/L, fT4 1.02 ng/dL [n.v. 0.93–1.7 ng/dL]). Her fasting C-peptide was 0.51 nmoL/L.

The patient was educated on diabetes management, including insulin injections and SMBG. Basal insulin was prescribed at a low dose of 0.1 UI/kg. She became pregnant just 2 months after the autoimmune T1DM diagnosis. Unfortunately, despite her optimal glucose control (HbA1c 31 mmol/mol), she experienced another miscarriage at GW 9. In the subsequent months, her blood glucose levels started to rise, requiring progressive insulin dosage adjustments. A few months after the second miscarriage, she became spontaneously pregnant (pre-conceptional HbA1c 30 mmol/mol) (timeline shown in Figure 1). She maintained a low dose of basal insulin during pregnancy (insulin detemir 4 UI in the evening, prior to bedtime), and her last HbA1c before delivery was 29 mmol/mol. At 38 GW, she delivered, after induction of labor, a healthy female baby (weight at birth 3,320 gr, 75th centile of growth according to INeS Chart, Apgar index 10 at 5′, 10 at 10′). After delivery, she discontinued insulin therapy. Three years later, she still has impaired fasting glucose, managed with a diet low in simple carbohydrates. Her most recent HbA1c was 36 mmol/mol, and fasting C-peptide was 0.53 nmoL/L.

## 5 Case 4

A 31-year-old white woman was referred to our clinic after a diagnosis of gestational diabetes. She had a family history of T1DM (her sister) and a known history of pre-gestational hypothyroidism, a previous spontaneous abortion, and previous surgery with Gamma Knife treatment for a pituitary adenoma.

The 75-gram OGTT performed at 24 + 6 GW was positive for GDM (time 0’ BG 5.28 mmoL/L, time 60’ BG 7.56 mmoL/L, and time 120’ BG 6.72 mmoL/L) (timeline shown in Figure 1). Good glucose control was achieved with nutritional therapy alone, but, due to the family history of T1DM, we recommended testing for beta-cell-specific autoantibodies. GADA were positive at a high titer (>2,000 U/mL), while ICA, IA-2 Ab, and ZnT8 Ab were negative. As macrosomia was suspected on ultrasound examination (fetal growth at the 96° centile according to Hadlock’s estimates), induction of labor was performed at 38 + 4 GW, which resulted in a vaginal delivery of a healthy male baby (weight at birth 3,670 g, 88th centile of growth according to INeS Chart, Apgar index 10 at 5’, 10 at 10’).

Postpartum glucose assessment was not available, and a few months after delivery, the patient became spontaneously pregnant for the second time. At 16 + 5 GW, she underwent a 75-gram OGTT, which was normal (time 0’ BG 4.56 mmoL/L, time 60’ BG 5.5 mmoL/L, and time 120’ BG 5 mmoL/L), and her HbA1c was 35 mmol/mol. At 26 + 5 GW, she underwent a second 75-gram OGTT, which was normal again (time 0’ BG 4.72 mmoL/L, time 60’ BG 5.94 mmoL/L, and time 120’ BG 7 mmoL/L). Pregnancy was complicated by polyhydramnios, and, after induction of labor at 38 + 3 GW, the patient gave birth to a female baby (weight at birth 3,450 g, 77th centile of growth according to INeS Chart, Apgar index 10 at 5′, 10 at 10′).

During her 5-year follow-up, the patient experienced the onset of overt T1DM, unfortunately with ketoacidosis. She was prescribed MDI therapy and glucose sensor monitoring, after appropriate education. At that time, her BMI was 20.9, and HbA1c was 100 mmol/mol. Autoimmunity for diabetes was retested, showing GADA > 250 u/ml, ZnT8 Ab 219.5 u/ml, and IA2-A 345.4 u/ml. Two years after the diagnosis of overt T1DM, the patient had a third pregnancy, which was managed with MDI and glucose sensor monitoring, achieving optimal glucose control. After induction of labor at 37 + 6 GW, she had a vaginal delivery and gave birth to a healthy neonate (birthweight 4,060 g, 100th centile, Apgar index 10 at 5’, 10 at 10’). No neonatal hypoglycemia or other postpartum adverse outcomes occurred.

## 6 Diabetes-related autoantibodies in gestational diabetes

Diabetes-related autoantibodies have been detected in pregnancies complicated by GDM. The prevalence varies widely according to the type of antibody, the population studied, and the assay used (6). The overall prevalence of diabetes-related autoimmunity in an unselected population of women with GDM ranges from <1 to 18% (7–11). The highest prevalence is reported in populations at high risk for T1DM, such as Sardinia (12) and Finland (13). All autoantibodies against pancreatic beta-cell antigens have been investigated in women with GDM. Islet cell autoantibodies (ICA) and anti-glutamic acid decarboxylase antibodies (GADA) have been more extensively studied and seem to be the most frequent in GDM (11, 14), while less data are available regarding the prevalence of antibodies against ZnT8 in GDM (15–17). Usually, only one antibody is detected in women with GDM, and the combination of two or more antibodies is rare. In the study by Luiro et al. (18), the women who tested positive for one antibody were 12% in the GDM population and 2.3% in controls, the women who tested positive for two antibodies were 2.6% in the GDM group and 0.3% in controls, and the women who tested positive for three antibodies were 2.3% in the GDM group and none in the control group.

Women with GDM and autoimmunity seem to have a peculiar clinical phenotype with few or no features of insulin resistance. They typically have a normal pre-pregnancy BMI, low waist circumference, low weight gain during pregnancy, and low fasting insulin levels (6, 10). In contrast with women with GDM without antibodies, they require insulin therapy more frequently (6, 8, 10). They are often younger and have a low prevalence of a family history of diabetes (8, 10).

Positivity for diabetes-related autoimmunity in GDM is associated with the subsequent development of T1DM, with the odds increasing in proportion to the number of positive autoantibodies. For example, in the study by Fuchtenbusch et al. (8), the risk for the onset of T1DM within 2 years after GDM increased from 17% in women who were positive for one antibody to 61% for those who were positive for two antibodies and to 84% for those who were positive for three antibodies. These results are also confirmed in other follow-up studies, in which the presence of autoantibodies was highly predictive of T1DM onset or impaired glucose metabolism, with follow-up periods of up to 8 years (19). In addition to the number of autoantibodies, other factors associated with a high risk of developing T1DM include age below 30 years, insulin therapy during pregnancy, and parity (6, 8, 18).

## 7 Onset of autoimmune diabetes in pregnancy

While there is significant literature on the presence of diabetes-related autoimmunity in pregnancies complicated by GDM, there are very few data on the onset of overt autoimmune T1DM during pregnancies (20, 21). This group of pregnancies is associated with a poor obstetrical prognosis. Buschard et al. (20) described the clinical course of 63 women who delivered between 1966 and 1980 and were diagnosed with overt autoimmune T1DM during pregnancy. In the majority of the cases, the diagnosis was made during the third trimester, when hyperglycemia due to GDM is usually detected. Mean fasting blood glucose at diagnosis was clearly elevated (15.6 ± 1.3 mmol/mol), and ketonuria was frequently present (81% of the women). The rate of preeclampsia was 9.5% and perinatal mortality was 6.3%, similar to those of women with pre-gestational diabetes, but higher than those of the healthy control group, where the rate of pre-eclampsia was 1.1% and perinatal mortality was 1.0%. On the other hand, congenital malformations were more frequent in the pre-gestational diabetes group than in the newly diagnosed women and the control group, maybe because hyperglycemia in the newly diagnosed women occurred at a certain point during pregnancy after conception, when organogenesis was completed or nearly completed. Nearly 20 years later, Wucher et al. (21) described a cohort of 21 women diagnosed with overt autoimmune T1DM during pregnancy and confirmed a high frequency of poor obstetrical outcomes (14 of 21 women). Among these cases, there were eight large-for-gestational-age neonates, four preterm deliveries, three neonatal hypoglycemic episodes, four admissions to the neonatal intensive care unit, and two fetal deaths associated with severe maternal hyperglycemia. The diagnosis of overt diabetes was made at a median of 26 GW, and more than 50% of the women had at least one risk factor for GDM. All women with newly diagnosed T1DM required insulin treatment during pregnancy.

The clinical course of autoimmune T1DM after pregnancy may vary, ranging from cases in which insulin therapy is continued after delivery to cases in which patients achieve insulin independence. In the French study by Wucher et al. (21), the remission period occurred in 85% of the population studied (median 180 days). In the cohort of Buschard et al. (20), 80% of patients had a remission period (median 256 days), although after a follow-up of 8 years, all patients were diagnosed with overt T1DM. In this cohort, the shortest remission period occurred in younger women with low parity, normal weight, and high blood glucose levels at the time of diagnosis. Remissions may be due to a restoration of insulin sensitivity after delivery (21).

Hyperglycemia due to T1DM in pregnancy is often misdiagnosed. At first, it may be diagnosed as GDM during routine screening, while antibody positivity may be detected only during postpartum follow-up due to the persistence of overt diabetes (21–23). Antibody positivity may be detected as part of the work-up for clearly elevated blood glucose values (20) or for the occurrence of negative obstetrical outcomes (21). Sometimes, autoimmune T1DM diagnosed during pregnancy is classified as latent autoimmune diabetes in adults (LADA), as they both share features such as insulin resistance, GADA positivity, slow progression, and adult onset (11, 21) (Table 2). GADA are the most frequent single islet cell-specific autoantibodies present in LADA (24), as it is in hyperglycemia in pregnancy. LADA usually is diagnosed after 30 years of age (24, 25). However, the onset of autoimmune diabetes during pregnancy can also happen before 30 years of age (21), even if the average age at first pregnancy is increasing, especially in Western countries. C-peptide levels slowly decrease in patients with LADA (26), but we do not have enough data on C-peptide levels during the follow-up of women with T1DM onset during pregnancy. Individuals with LADA typically do not require insulin for at least 6 months after diagnosis (24), but women with hyperglycemia during pregnancy do require insulin, which remains the safest therapy option. Nonetheless, T1DM has phenotypic variability, and the unequivocal definition of LADA is still a matter of debate (24).

**Table 2:** Comparison between autoimmune diabetes with onset in pregnancy and LADA.

	Autoimmune diabetes with onset in pregnancy	LADA
GADAb positivity	Most frequent	Most frequent
Age of onset	Any age within the fertile years	>30 years
C-peptide trend	Not enough data	Slow decline
Insulin therapy	As soon as possible according to glucose levels	At least after 6 months

GADAb, anti-GAD antibodies; LADA, Latent autoimmune diabetes in adults.

Hyperglycemia in pregnancy presents in a wide variety of forms. Diabetes-related autoimmunity affects approximately 10% of pregnancy cases complicated by GDM (range 1–18%), conferring a high risk of adverse obstetrical outcomes and progression to overt T1DM in the years after delivery. In this context, a timely diagnosis is important for the appropriate management of pregnancy and clear planning of follow-up visits after delivery.

There are no guidelines specifying who to test for diabetes-related autoimmunity when hyperglycemia is detected during pregnancy, and variable clinical phenotypes can make diabetes classification difficult. Known predisposing factors for autoimmunity positivity in women with hyperglycemia during pregnancy include normal or low pre-pregnancy weight, younger age, low weight gain during pregnancy, the need for insulin therapy, and no family history of T2DM (8, 10, 21). In our series, three of the four women had a family history of T2DM and autoimmune diseases, and two patients had siblings affected by T1DM. According to our experience, a family history of autoimmune disease—especially T1DM—should be considered one of the major risk factors for the onset of T1DM during pregnancy in women with documented hyperglycemia. In our case series, all the women had a normal weight. The age at conception reflected the median age of first pregnancy in our population (above 30 years of age). Insulin therapy was necessary in more than half of our patients. Considering our center’s experience, albeit with a small sample size, we suggest that suspicion of T1DM during pregnancy should arise if women have two or more of the following conditions: a family history of T1DM, normal or low pre-pregnancy BMI, need for insulin therapy, higher requirement for rapid-acting insulin compared to long-acting insulin, and a family or personal history of autoimmune disease.

The available data in the literature do not allow for a direct comparison of pregnancy outcomes between new-onset and established T1DM during pregnancy. The reports by Buschard et al. (20) and Wucher et al. (21) highlight rates of adverse pregnancy outcomes comparable to those seen in pregnancies complicated by pre-gestational diabetes. In particular, Wucher et al. (21) reported two fetal deaths associated with severe maternal hyperglycemia. In our small sample, we did not report an increased rate of adverse pregnancy outcomes, as in pre-gestational diabetes (i.e., lower rate of macrosomia), but it is worth noting that all women were managed by an experienced multi-disciplinary team to limit complications and decide the appropriate timing of delivery (i.e., changes of AFI in Case 2). Moreover, none of our cases had elevated glucose or ketonemia, which could acutely threaten the life of the fetus. We can conclude that pregnancy complicated by new-onset T1DM is a high-risk pregnancy, similar to pregnancies in women with pre-gestational diabetes, although a multi-disciplinary approach and timely monitoring may reduce adverse outcomes.

The onset of T1DM during pregnancy may have a significant psychological impact on women and their families; therefore, they should be offered psychological support during pregnancy and after delivery. Unfortunately, due to the retrospective nature of our study, we did not have data on patient-relevant outcomes and long-term follow-up after delivery.

In the real world, universal screening for beta-cell–specific autoantibodies in every pregnant woman with hyperglycemia may not be affordable on a large scale. However, it could be considered in a limited number of pregnancies with specific risk factors or in settings in which only a small quantity of serum (27) or saliva is required (28). It is important to identify women with autoimmunity, as they can be offered, after delivery, treatment with drugs that may delay the overt onset of T1DM (29), as well as adequate information and follow-up.

Further research is needed to better characterize pregnancies that deserve a timely identification, so that affected women can receive appropriate care and follow-up in specialized referral centers.

## Data Availability

The raw data supporting the conclusions of this article will be made available by the authors, without undue reservation.
